# The Effects of Lifestyle Intervention Using the Modified Beliefs, Attitude, Subjective Norms, Enabling Factors Model in Hypertension Management: Quasi-Experimental Study

**DOI:** 10.2196/20297

**Published:** 2021-09-24

**Authors:** Resti Tito Villarino, Christopher Asuncion Arcay, Maria Concepcion Temblor, Maureen Lorence Villarino, Rosita Bagsit, Lanndon Ocampo, Paquito Bernard

**Affiliations:** 1 Cebu Technological University, Moalboal Campus Cebu Philippines; 2 National Research Council of the Philippines Manila Philippines; 3 Mater Dei College Tubigon, Bohol Philippines; 4 University of San Agustin Iloilo City Philippines; 5 Cebu Technological University, Main Campus Cebu City Philippines; 6 Université du Québec à Montréal Montréal, QC Canada; 7 Research Center of the University Institute in Mental Health of Montreal Montréal, QC Canada

**Keywords:** hypertension, BASNEF, blood pressure, medication adherence

## Abstract

**Background:**

Hypertension is a serious health issue and a significant risk factor for cardiovascular disease and stroke. Although various health education models have been used to improve lifestyle in patients with hypertension, the findings have been inconsistent.

**Objective:**

This study aims to assess the effects of a lifestyle intervention program using a modified Beliefs, Attitude, Subjective Norms, Enabling Factors (BASNEF) model among nonadherent participants with hypertension in managing elevated blood pressure (BP) levels.

**Methods:**

This study reports a quantitative quasi-experimental research work, particularly using a repeated-measures design of the within-subjects approach on the 50 nonadherent patients who received a diagnosis of essential hypertension in Cebu, Philippines. The research participants received 5 sessions of training based on a modified BASNEF model. An adherence instrument was used as an evaluation platform. The first phase gathers participants' relevant profiles and background, and the final phase gathers participants' systolic BP, diastolic BP, heart rate, and adherence scores.

**Results:**

The results indicate that the phase 1 mean systolic readings (146.50, SD 19.59) differ significantly from the phase 4 mean systolic readings (134.92, SD 15.24). They also suggest that the lifestyle intervention based on session III or phase IV behavioral intention in the BASNEF model microgroup sessions positively affects BP readings among the research participants.

**Conclusions:**

This study has established that the BASNEF model approach can be a good BP management technique.

## Introduction

### Background

The third goal (ie, goal 3, to ensure healthy lives and promote well-being for all ages) of the United Nations Sustainable Development Goals targets reducing premature deaths from noncommunicable diseases by one-third through prevention and treatment by the year 2030 [1]. One crucial health dilemma worldwide is the prevalence of hypertension. Hypertension is defined as systolic blood pressure (BP) elevated to more than 140 mm Hg or diastolic BP less than 90 mm Hg [2] and is one of the factors that increase mortality in both high-income and low-to-middle–income countries. According to the World Health Organization, hypertension accounted for 12% of global deaths in 2013 [3]. The prevalence of hypertension in adults (>18 years) is 22% [4].

Hypertension is the most common cardiovascular disease, often resulting in stroke, heart attack, kidney disease, and aneurysm [5]. Patients with hypertension do not meet the existing BP goal set in international guidelines [6]. Target levels were set to measure the BP and to determine whether readings belong to the standard category. In addition to the BP readings, the heart rates (HRs) were also measured. Another set of categories was also established to determine whether HR readings belong to the standard category [7]. The etiology of hypertension is unknown in the current literature. Some potential factors have been identified, including obesity, diet, and physical activity [8]. Hypertension cannot be treated under normal conditions, but it can be managed through medication as the primary management scheme. More than 100 different medicines with proven efficacy are available; most have untoward side effects, and many are formulated for maintenance [9].

The traditional approach in managing hypertension involves following health guidelines to prevent adverse health issues. Despite these guidelines, encouraging patients to modify their lifestyle over time remains a considerable challenge in hypertension management. How health education is implemented is crucial in helping patients change their lifestyle over the long term [10]. The World Health Organization considers lifestyle as the regular patterns of behaviors resulting from interactions among characteristics, relationships, milieu, and economic circumstances [11]. Lifestyle plays an essential role in hypertension development [12]. The current literature has reported particularly critical lifestyle factors, including nutrition, exercise, stress, smoking, weight management, sleep, and rest. The Seventh Report of the Joint National Committee on Prevention, Diagnosis, Evaluation, and Treatment of hypertension considers inadequate attention to a lack of social coverage of health education as the most significant obstacle to health education [13].

Various literature models were offered as supportive behavioral change tools and current sociocultural contexts [14,15]. Among them is the Beliefs, Attitude, Subjective Norms, Enabling Factors (BASNEF) model, which is a systematic and comprehensive tool “to study behaviors and plans to change them and to define the factors effective on individuals’ decision-making” [16]. The underlying concept behind the BASNEF model is that individuals develop a new behavior when they feel that the behavior is helpful to them [16]. The assessment process individuals implement to understand the efficacy of behavior contributes to their attitudes about their behavior. The direct implication of this concept is that key individuals in one’s life can influence one’s decision for new behavior, which in turn acts as either a facilitator or an inhibitor. Because beliefs dictate the subjective norms of individuals, the convergence of social expectations contributes in the development of decision-making associated with achieving new behavior. On the contrary, factors such as ability, resources, and expense, among others, can help turn a purpose into efficient action. These conditions usually exist before a behavior occurs [14].

As a healthy lifestyle is widely believed to be a critical factor in reducing disease incidence, severity, and complications, particularly in hypertension, the BASNEF model addresses a significant gap. As a framework, the BASNEF model considers environmental and social norms in changing behavior, in addition to the knowledge and attitude of patients. A prerequisite for an effective health education model is an understanding of the factors underlying the behavior of a person. Conceptually, the BASNEF model is a simplified behavioral understanding approach based on the Predisposing, Reinforcing, and Enabling Constructs in Educational/Environmental Diagnosis and Evaluation model and value expectation theory. Applying the BASNEF strategy involves assessing the group outlook for behavior with adequately defined actions. In addition to adequately defined actions, it is also essential to pay attention to the facilities and knowledge that a motivated person needs to ensure that all enabling factors are available for a motivated person. With these factors in place and a good understanding of the immediate community, it is possible to use the BASNEF model to design health education interventions.

Few studies have explored the BASNEF model to improve the lifestyle of patients with hypertension. The model has been used to develop lifestyle changes in different domains, including investigations for several interventions [13,17], self-monitoring [18], and lower BP [19], and cesarean section rates among pregnant women. Various studies have demonstrated that enhancing variables such as family and support [20], attitude, and subjective norms [21], which are elements of the BASNEF model, can substantially anticipate health habits and long-term adherence treatments. Clinical findings have shown that health education and fitness programs [22] and a holistic lifestyle change [23] in patients with hypertension may significantly improve hypertension, diet, weight, and physical activity. In comparison, several studies have indicated that health education [24] and lifestyle education [25] can improve the understanding of patients with hypertension. However, BP cannot be reduced [24]. A study revealed that nurse-led therapy (ie, trying to affect patient subjective norms) did not affect outpatient BP and antihypertensive drug treatments in patients at elevated cardiovascular disease risk [26].

In patients with hypertension, health education has been shown to have a significant impact on self-care behaviors. The prevalence of precautionary measures via health education is a robust tool for healthier lifestyles while safeguarding from complications of hypertension. It was also found that the use of these preventive procedures in educating self-care behaviors in patients with hypertension without a holistic educational structure is less vital than conventional teaching, considering the long history of design and educational structures in global health systems and given that these approaches are preferred educational instruments [13]. The training provided is not successful without the use of educational models or rational processes to transform behavior. Although parameters in awareness and attitude and their influence on behavior are significant, during care and compliance with patients receiving nutritional therapy, other significant factors, such as personal abilities and milieu, affect the conduct and behavior of patients. Thus, it can be inferred that using important constructs and health education models to produce better outcomes would yield outputs that are more successful. Although the BASNEF model has been implemented in hypertension management, to the best of our knowledge, no work has been reported on measuring the effectiveness of health education programs in managing hypertension in the context of the BASNEF. Consequently, this study was conducted to examine the effects of a lifestyle intervention program using the BASNEF model among nonadherent hypertensive respondents.

### Objectives

This study aims to determine if there is significant evidence to support the following research hypotheses (RH):

1. The different phases of medication significantly differ in systolic BP readings (RH1), diastolic BP readings (RH2), and HR readings (RH3).
2. Sex classifications of the participants differ significantly in systolic BP readings (RH4), diastolic BP readings (RH5), HR readings (RH6), and Morisky Scale (MS) scores (RH7).
3. Age classifications of the participants differ significantly in systolic BP readings (RH8), diastolic BP readings (RH9), HR readings (RH10), and MS scores (RH11).
4. Medication adherence (ie, MS scores) of the participants differs significantly in systolic BP readings (RH12), diastolic BP readings (RH13), and HR readings (RH14).
5. The age of the participants significantly relates to systolic BP readings (RH15), diastolic BP readings (RH16), HR readings (RH17), and MS scores (RH18).
6. Medication adherence (ie, MS scores) of the participants significantly relates to systolic BP readings (RH19), diastolic BP readings (RH20), and HR readings (RH21).

## Methods

### Methods and Materials

This study is a quantitative quasi-experimental research work, particularly using a repeated-measures design of the within-subjects approach [27-29]. A study in the Philippines on 50 nonadherent patients diagnosed with essential hypertension was reported. The research participants received 5 sessions by phase in the context of a modified BASNEF model. A Morisky Medication Adherence instrument was used. The first phase of the program involved distributing the demographic questionnaire, including the age and sex of the participants. The final phase focused on the evaluation of the program. Self-BP and HR monitoring were performed daily in the morning and recorded by the patient or significant other using a standard digital sphygmomanometer (ie, Docteurs Choice Arm-cuff Digital Blood Pressure Monitor) that was provided to them for the duration of the program. Monitoring was performed after breakfast, before taking antihypertensive maintenance medications. BP and HR readings were collected every Friday, and the average readings were reported. The time frame of the program was 10 months. The University Research Ethics Committee of Cebu Technological University approved the design of the research, protocols, and informed consent process.

### Study Population

This study was conducted on 120 nonadherent patients diagnosed with stage 1 or stage 2 hypertension listed in the Rural Health Unit-Hypertension Club. The participants are currently living in a mountain village in the municipality of Moalboal, Cebu (Philippines). The sampling design followed the approach in Arani et al [12]. A set of inclusion criteria was established to determine the participants of the study from the 120 listed patients. Of the initial 120 patients, 20 failed to meet the inclusion criteria, and 50 did not agree to participate. A total of 50 nonadherent patients provided their consent to participate in the study. No participant was excluded owing to withdrawal from the study, absence at >1 session, or hospitalization.

### Instrument

In this study, the MS determines the level of medication adherence of the participants with hypertension. MS is a standardized measure intended to measure the risk of nonadherence to medication [20,30]. This method is widely used in domain literature. It is used for various illnesses, such as high BP, hyperlipidemia, asthma, and HIV. The results are based on answers of patients to 4 questions, which are answered by a yes or a no.

### The Lifestyle Intervention Program

The intervention was given every Friday afternoon in 5 sessions after collecting their BP and HR readings. Every session had a design that integrated various aspects of a lifestyle change program. Participants received printed materials containing Microsoft PowerPoint slides included in the sessions expressed in the local dialect (ie, Cebuano) for reference. The relevance and internal validity of the educational materials draw parallel to the work of Arani et al [12] and Villarino et al [31]:

1. Phase 1 (month 1): This phase covers activities such as orientation to the session design, signing of the informed consent document, finishing the research tool (ie, MS), and carrying out the baseline measurements (ie, BP and HR readings).
2. Session I, phase 2 (month 2): This phase aims to enhance the knowledge and transform the behavior, attitudes, and beliefs of the participants based on the BASNEF model. It also provides 45-60 min/week lecture on hypertension (eg, the definition of hypertension, its causes, and several contributing factors).
3. Session II, phase 3 (month 3): This phase discusses the cumulative and salient effects of high BP and the consequences of smoking, alcohol, and caffeine. It also elucidates the side effects of antihypertensive drugs.
4. Session III, phase 4 (month 4): This phase highlights the behavioral intention of the BASNEF model (ie, via microgroups). It aims to educate the participants on what action is anticipated and how to do it (eg, practical tips on keeping the ideal weight considering the BMI, methods for reducing salt consumption, and practical stress management methods).
5. Session IV, phase 5 (month 5): This phase elucidates subjective norms. It includes a session for individuals who would help improve the lifestyle of the participant and thus help reduce hypertension, such as their partner and children, in a way that explains their role in behavioral modification and hypertension management.
6. Session V, phase 6 (month 6): This phase emphasizes the enabling factors. At meetings, all participants were provided with a pamphlet to keep the training fluid. The participants were informed of how to use health care center services and access the necessary treatment.
7. Phase 7 (month 7): This phase includes the necessary evaluation activities, such as reviewing the previous education sessions, conducting the posttest and BP readings, and taking a post–lifestyle intervention program for the participants.

### Statistical Analysis

Categorical variables, particularly the sex and age of participants, were expressed as frequencies and percentages. Continuous variables, such as systolic BP, diastolic BP, HR readings, and MS scores, were defined as mean (SD). Repeated-measures one-way analysis of variance (ANOVA) was used for testing RH1 to RH14, except for RH4 to RH7, in which a 2-tailed independent *t* test was used. When an RH was rejected (eg, RH4, RH5, and RH8), a Tukey post hoc test was used to identify the source of the significant differences. To test the association of age and BP and HR readings, and MS scores and MS scores and BP and HR readings (ie, RH15 to RH21), the Pearson product-moment correlation test was used. Correlation coefficients (*r*) and *P* values were reported. Finally, the 1-way ANOVA test was used to test the differences in systolic BP, diastolic BP, and HR readings according to MS scores. The significance level of all tests was set at α=.05. All analyses were performed using R (programming language; R Foundation for Statistical Computing).

## Results

### Profiles of the Participants

The distribution of research participants in terms of sex and age are presented in Table 1. More than three-fourths of the participants were female (39/50, 78%). The ages of these participants also show a relatively bimodal distribution, with those in the age groups of 40-49 and 70-79 years having the highest frequencies.

**Table 1:** Profile of the participants (N=50).

Profile		Frequency, n (%)
**Sex**		
	Female	39 (78)
	Male	11 (22)
**Age (years)**		
	80-89	4 (8)
	70-79	13 (26)
	60-69	8 (16)
	50-59	7 (14)
	40-49	12 (24)
	30-39	6 (12)

### BP and HR Readings

Table 2 shows the highest BP readings of the participants, with a mean of 146.50/84.6 in phase 1 of the study. Note that phase 1 denotes the baseline BP readings of participants with hypertension before they received interventions via health education. The lowest BP readings of the participants with a mean of approximately 135/80 were recorded in phase 4 of the study.

**Table 2:** Blood pressure and heart rate readings of the participants.

Phases	Blood pressure readings^a^			Heart rate readings^b^	
	Systolic, mean (SD)	Diastolic, mean (SD)	Interpretation	Heart rate, mean (SD)	Interpretation
1	146.50 (19.59)	84.60 (12.59)	Hypertension stage 2	77.76 (11.65)	Normal
2	136.64 (18.42)	80.36 (11.83)	Hypertension stage 1	76.92 (10.71)	Normal
3	136.80 (16.37)	79.26 (9.90)	Hypertension stage 1	76.08 (9.93)	Normal
4	134.92 (15.24)	78.76 (10.81)	Hypertension stage 1	77.94 (12.43)	Normal
5	137.88 (19.18)	79.96 (11.24)	Hypertension stage 1	79.16 (12.01)	Normal
6	136.86 (15.14)	79.58 (10.76)	Hypertension stage 1	77.06 (11.32)	Normal

^a^Mean systolic blood pressure, 138.27 (SD13.31); mean diastolic blood pressure, 80.42 (SD 7.97).

^b^Mean heart rate, 77.49 (SD 7.70); interpretation: normal.

As presented in Table 2, the HR readings of the participants were highest in phase 5, with a mean of 79.16, and the lowest mean readings of 76.08 were recorded in phase 3. Increased HR is associated with high BP, increased risk of hypertension, and increased cardiovascular disease risk in patients with hypertension [32].

### Differences in Systolic BP, Diastolic BP, and HR Readings, and MS Scores

A summary of the results of the tests for RH1 to RH14 (numerical values and descriptive entries on the differences in systolic BP, diastolic BP, and HR readings based on the phases of medication, sex, age, and MS scores) is presented in Table 3.

Using repeated-measures ANOVA, all *P* values of the systolic BP (*P*=.80), diastolic BP (*P*=.90), and HR (*P*=.46) readings exceeded the level of significance of .05, based on the stages of medication. These results lead to the nonrejection of the null hypotheses of no significant differences in the systolic BP, diastolic BP, and HR readings of the participants based on all medication phases. The *P* values of the systolic readings (*P*=.02) and the diastolic readings (*P*<.001) were less than the significance level of .05, based on sex, after using an independent *t* test. This implies rejecting the null hypothesis of no significant difference in systolic and diastolic BP readings. However, because the *P* values of the HR readings (*P*=.56) and MS scores (*P=*.25) of the male and female participants exceeded the level of significance of .05, the null hypotheses of no significant differences were not rejected.

The *P* value of the systolic BP readings (*P*<.001) was less than the significance level of .05, after using 1-way ANOVA. The null hypothesis of no significant difference was rejected, which led to the RH’s support (RH8). In this study, the systolic BP readings of the participants differed significantly when grouped according to their age classifications. After using the Tukey post hoc test to determine which age classifications differ from others, it was found out that those in the age group 30-39 years had significantly lower average systolic BP readings of 121.72 compared with the rest of the age groups that did not differ. In contrast, after the 1-way ANOVA test, as the diastolic BP readings (*P*=.28), HR (*P*=.24) readings, and MS scores (*P*=.29) had *P* values greater than the level of significance of .05, the null hypotheses of no significant differences were not rejected. The *P* values of the systolic BP (*P*=.08), diastolic BP (*P*=.11), and HR (*P*=.59) readings were higher than the significance level of .05, after a 1-way ANOVA test. This leads to the null hypothesis that there is no significant difference (ie, RH12, RH13, RH14). In Table 3, the evidence was not enough to support the RHs that the systolic BP (RH1), diastolic BP (RH2), and HR (RH3) readings significantly differ based on the phases of medication adherence. This finding suggests that the participants had statistically the same systolic BP, diastolic BP, and HR readings in the phases of medication adherence. Reductions or increases that were observed in all the BP and HR readings from 1 phase to the next were insignificant. Sufficient evidence supports the RHs that both systolic BP (RH4) and diastolic BP (RH5) readings differ based on sex. This result implies, more specifically, that the male participants had a significantly higher mean systolic BP of 146.61 and mean diastolic BP of 88.62 compared with the systolic mean of 135.92 and diastolic mean of 78.11 of the female participants. The evidence did not support the RHs that the HR readings (RH6) and MS scores (RH7) differ according to sex. Thus, the male and female participants had the same HR readings and medication adherence as indicated by the MS scores. The results did not support the RHs that diastolic BP readings (RH9) and HR readings (RH10), together with MS scores (RH11), differ according to age. These results imply that diastolic BP and HR readings and MS scores were statistically the same according to age.

**Table 3:** Summary of inferences on systolic BP^a^, diastolic BP, and HR^b^ readings, and MS^c^ scores based on the phases of medication, sex, age, and MS scores.

Comparison bases			RH^d^		Critical value^e^		Test value		*P* value		Decision on H0		Difference
**Phases of medication**													
	Systolic BP readings	RH1		2.42		0.41		.80		Not rejected		Not significant	
	Diastolic BP readings	RH2		2.42		0.27		.90		Not rejected		Not significant	
	HR readings	RH3		2.42		0.90		.46		Not rejected		Not significant	
**Sex**													
	Systolic BP readings	RH4		2.11		2.59		.02		Rejected		Significant	
	Diastolic BP readings	RH5		2.10		4.85		<.001		Rejected		Significant	
	HR readings	RH6		2.13		0.59		.56		Not rejected		Not significant	
	MS scores	RH7		2.08		1.18		.25		Not rejected		Not significant	
**Age (years)**													
	Systolic BP readings	RH8		2.43		5.10		<.001		Rejected		Significant	
	Diastolic BP readings	RH9		2.43		1.30		.28		Not rejected		Not significant	
	HR readings	RH10		2.43		1.40		.24		Not rejected		Not significant	
	MS scores	RH11		2.43		1.28		.29		Not rejected		Not significant	
**MS scores**													
	Systolic readings	RH12		2.58		2.21		.08		Not rejected		Not significant	
	Diastolic readings	RH13		2.58		2.02		.11		Not rejected		Not significant	
	HR readings	RH14		2.58		0.71		.59		Not rejected		Not significant	

^a^BP: blood pressure.

^b^HR: heart rate.

^c^MS: Morisky Scale.

^d^RH: research hypothesis.

^e^The critical values used were *F* values except those of sex, which used *t* values. The significance level was set at *P*=.05.

### Differences in Systolic BP, Diastolic BP, and HR Readings Per MS Scores

The *P* values of the systolic BP, diastolic BP, and HR readings are provided in Table 4.

The *P* values of the systolic BP (*P*=.08), diastolic BP (*P*=.11), and HR (*P*=.59) readings shown in Table 4 were higher than the significance level of .05 after the one-way ANOVA test. This leads to the nonrejection of the null hypothesis of no difference. The findings presented in Table 4 do not support the RHs advanced in this study that the systolic BP (RH12), diastolic BP (RH13), and HR (RH14) readings of the participants significantly differed based on MS scores. Hence, all BP and HR readings were statistically the same when grouped on the basis of medication adherence through MS scores. This result suggests that they had the same systolic BP, diastolic BP, and HR readings regardless of the medication adherence of the participants.

**Table 4:** Significance of differences in systolic BP^a^, diastolic BP, and heart rate readings based on Morisky Scale scores.

	Critical *F* value (*df*)	Computed *F* value (*df*)	*P* value^b^	Decision on H0	Difference
Systolic BP readings	2.58 (4)	2.21 (45)	.08	Not rejected	Not significant
Diastolic BP readings	2.58 (4)	2.02 (45)	.11	Not rejected	Not significant
Heart rate readings	2.58 (4)	0.71 (45)	.59	Not rejected	Not significant

^a^BP: blood pressure.

^b^Significance level *P*=.05.

### Relationships in Systolic BP, Diastolic BP, HR Readings Per Age, and MS Scores

Table 5 presents the numerical values and descriptive entries on the significance of relationships between the age of the participants and the BP and HR readings and the MS scores, and between the MS scores and BP and HR readings after using the Pearson product-moment correlation test. The systolic BP readings had a *P* value of .002, which was lower than the level of significance of .05. This leads to the rejection of the null hypothesis and sufficient evidence to support a significant relationship claimed in RH15.

**Table 5:** Significance of relationships between the readings with MS^a^ scores and age and between the readings and MS scores.

Relationship bases			Correlation coefficient (*r*)		*P* value		Decision on H0		Relationship
**Age**									
	Systolic	0.42		.002		Rejected		Significant	
	Diastolic	0.10		.49		Not rejected		Not significant	
	Heart rate	0.11		.43		Not rejected		Not significant	
	MS scores	0.25		.08		Not rejected		Not significant	
**MS scores**									
	Systolic	0.26		.07		Not rejected		Not significant	
	Diastolic	0.21		.14		Not rejected		Not significant	
	Heart rate	0.10		.51		Not rejected		Not significant	

^a^MS: Morisky Scale.

In contrast, because the *P* values of diastolic BP (*P*=.49) and HR (*P*=.43) readings and MS scores (*P*=.08) were more than the level of significance of .05, the null hypotheses of no relationship were not rejected. In addition, the *P* values of systolic BP (*P*=.07), diastolic BP (*P*=.14), and HR (*P*=.51) readings exceeded the level of significance of .05; therefore, the null hypotheses of no significant relationship were not rejected. Thus, the evidence failed to support the RHs that MS scores were significantly related to systolic BP (RH19), diastolic BP (RH20), and HR (RH21) readings. Hence, though this study found a significant relationship between systolic BP and age, it failed to find any effect of medication adherence through the MS scores on the systolic and diastolic BP readings and HR readings of the participants. However, low compliance to antihypertensive drugs is standard and contributes to poor BP control and adverse effects [32]. There is a lack of understanding of how patient-specific challenges affect poor adherence to medication and how successful approaches can be aimed at overcoming obstacles and enhancing adherence behavior in hypertensive adults.

## Discussion

### Principal Findings

This study assessed the effects of a lifestyle intervention program on nonadherent patients with hypertension using a modified BASNEF model. The results suggest that the lifestyle intervention based on session III or phase IV behavioral intention in the BASNEF model microgroup sessions positively affects BP readings among the research participants. The descriptive results indicate that the BP readings of the population under consideration were not significantly different from their MS scores.

In the last 3 months of the implementation of the program, our study found significant modifications in systolic and diastolic BP and improvements in all behavioral factors. Participants had reduced dietary sodium intake, had taken their maintenance antihypertensive medications if available, and if budget warranted, and had limited their alcohol and cigarette consumption. Our findings indicate that lifestyle change interventions have effectively decreased BP levels by having participants take steps such as reducing salt consumption, taking prescribed medicines, and limiting alcohol and cigarette consumption [33,34]. This agrees with some findings in the literature, such as the study conducted by Englert et al [35], which found substantial decreases in cholesterol, low-density lipoprotein, triglycerides, blood glucose, and BP levels, and weight loss in a group-based risk prevention plan. Repeated measures revealed that the program substantially decreased systolic and diastolic BP during the 10-month program implementation and a follow-up visit 3 months later. Previous studies found that lifestyle modifications could enhance BP regulation and reduce the risk of chronic illnesses [13,36].

Our results are also consistent with studies that used the BASNEF model to manage anxiety disorders in patients with hypertension [37]. Our study finds that a BASNEF-based program may improve all aspects of patient lifestyle by increasing awareness, enhancing patient beliefs and attitudes, and altering social norms through the participation of family members of patients. In addition, Baghianimoghadam et al [5] and Arani et al [12] argued that the lifestyle intervention program anchored on the BASNEF model has considerable advantages and significantly increases medication adherence in patients with hypertension. However, a few studies have indicated that behavioral approaches in patients with unstable angina [23] could not modify their lifestyle (ie, the prevalence of smoking, drug abuse, and mental health). Khavoshi et al [2] pointed out that lifestyle, social, and psychological well-being in older adults could not be influenced by educational interference using a self-belief model. Many habits, such as avoiding smoking, have a complicated structure, and many social and personality variables are affected. Therefore, improving knowledge and understanding of the population cannot influence these habits. However, the BASNEF model recognizes several factors, including intelligence and understanding, attitudes, cultural values, and milieu and social influences [13,23,38]. A wide range of people, such as families, friends, and other essential persons, can affect the understanding of social and psychological well-being of the patients. Thus, the BASNEF model would affect habits and activities of individuals and affect their psychosocial well-being, which family and other significant people influence [12]. This study found that designing and implementing theme-driven programs that merit milieu is more effective than the current health promotion programs that acknowledge the knowledge of patients, attitudes, and beliefs without regard to the factors that influence their behavior. This research also found that lifestyle interventions for managing hypertension that need consistent attention to medication adherence, diet, and behavioral management of the individual must involve the patients and health care workers, family, and the people who have impact on the behavior of patients.

### Conclusions

This study established that the BASNEF model approach can be an effective BP management technique. A significant change in BP was observed in sessions III or phase IV of the program, where behavioral intention in the BASNEF model (ie, microgroups) was implemented. The results of this study are relevant for patients with hypertension without comorbidities such as diabetes mellitus and physical or mental disorders. For future work, a longitudinal study may be conducted to determine the significant difference between the BP and HR readings among the respondents and emphasize the essentiality of medical adherence in managing hypertension using health education models.

## Abbreviations

ANOVA: analysis of variance
BASNEF: Beliefs, Attitude, Subjective Norms, Enabling Factors
BP: blood pressure
HR: heart rate
MS: Morisky Scale
RH: research hypothesis
